# Constitutive and UV-B modulated transcription of Nod-like receptors and their functional partners in human corneal epithelial cells

**Published:** 2008-08-29

**Authors:** Szilvia Benko, Jozsef Tozser, Gabriella Miklossy, Aliz Varga, Janos Kadas, Adrienne Csutak, Andras Berta, Eva Rajnavolgyi

**Affiliations:** 1 Institute of Immunology, Medical Health and Science Center, University of Debrecen, Debrecen, Hungary; 2 Department of Biochemistry and Molecular Biology, University of Debrecen, Debrecen, Hungary; 3 Ophthalmology, Medical Health and Science Center, University of Debrecen, Debrecen, Hungary; 4 InnoTears Ltd, Debrecen, Hungary

## Abstract

**Purpose:**

To determine the transcription pattern of Nod-like receptors (NLRs) and inflammasome components (apoptosis-associated speck-like protein containing a CARD [ASC], CARD inhibitor of NFkB-activating ligands [Cardinal], and caspase﻿-﻿1) in human corneal epithelial cells obtained from healthy individuals undergoing photorefractive keratectomy and in immortalized human corneal epithelial cells (HCE﻿-﻿T).

**Methods:**

Human corneal epithelial cells were taken from the eyes of healthy individuals by epithelial ablation for photorefractive keratectomy (PRK). The SV-40 immortalized human corneal epithelial cell line (HCE-T) was cultured. mRNA obtained from the cells was reverse transcribed and subjected to quantitative real-time polymerase chain reaction (PCR) measurements. Protein obtained from HCE-T cells was studied using the western blot technique. HCE-T cells were irradiated by UV-B light or treated with ultrapure peptidoglycan, and the effects were studied at the mRNA and protein level while the supernatant of the cells was tested for the presence of various cytokines by using enzyme-linked immunosorbent assay (ELISA) methods.

**Results:**

mRNA levels of the studied proteins in the primary cells of the donors were similar in most cases. The transcription of *Nod1*, *Nod2*, *NLRX1*, *Nalp1*, and *Cardinal* was similar in the two cell types. While the expression of *Nalp3* and *Nalp10* was higher in HCE-T cells, *ASC* and *caspase-1* showed higher transcription levels in the primary cells. *NLRC5* and *Nalp7* were hardly detectable in the studied cells. Functionality of the Nod1/Nod2 system was demonstrated by increased phosphorylation of IkB upon Nod1/Nod2 agonist ultrapure peptidoglycan treatment in HCE-T cells. While UV-B irradiation exerted a downregulation of both *Nalp* and *Nod* mRNAs as well as those of inflammasome components in HCE-T cells, longer incubation of the cells after exposure resulted in recovery or upregulation only of the Nalp sensors. At the protein level, we detected a short isoform of Nalp1 and its expression changed in a similar way as its RNA expression, but we could not detect Nalp3 protein. Among the studied cytokines, only IL-6 was detected in the supernatant of HCE-T cells. Its constitutively secreted level increased by only twofold after 24 h of UV-B irradiation.

**Conclusions:**

Based on our experiments, UV-B irradiation appears to exert an immunosilencing effect on the HCE-T cells by downregulating most of the sensor molecules as well as the components of the inflammasomes. Expression profiling of corneal epithelial cells suggested that the HCE-T cells may not serve as a good model for Nalp3 or Nalp1 inflammasome studies but it may be better suited for studies on the Nod1/Nod2 systems.

## Introduction

Corneal epithelial cells are non-keratinized, stratified squamous cells that not only provide a physical barrier, but through sensing invading pathogens, they also contribute to the first line of defense mediated by innate immunity [1]. Epithelial cells of the eye are in continuous contact with nonpathogenic microbes that do not elicit immune responses under physiological conditions. However, damage, infection, trauma, or injury of the eye may elicit inflammatory immune responses even against nonpathogenic, commensal bacteria.

Besides protecting the eye from microbial infection, corneal epithelial cells are also important in the defense of the retina from various stresses such as ultraviolet (UV)-induced photodamage. The cornea as the most outer layer of the eye absorbs a substantial amount of UV-B radiation. The only acute clinical effect of UV radiation of the eye is photokeratitis (snow blindness or welder’s flash), a painful but transient inflammatory condition caused by UV-C and UV-B induced damage of the cornea, which typically appears 6﻿–﻿12 h after exposure and resolves within 48 h [2]. Following UV-B irradiation, intracellular changes in the fine structure of cells, accumulation of self-aggregates, appearance of neoantigens, and adsorption of microorganisms may contribute to this reaction, and in the long term, UV-B irradiation may increase the susceptibility to pathogens responsible for the development of ocular pathological disorders.

Nod-like receptor (NLR) family proteins have recently been shown to represent an intracellular pathogen sensing system in mammals. These cytosolic proteins are structurally similar to resistance (R) proteins of plants that are involved in disease resistance against pathogenic infection [3,4]. Furthermore, they exhibit structural and functional similarities to toll-like receptor (TLR) family proteins and thus belong to a conserved recognition system of innate immunity [5]. NLRs act as sensors of pathogens as part of specialized cytosolic protein complexes (Nalp inflammasome, Nod-signalosome) formed by association with the adaptor molecules like apoptosis-associated speck-like protein containing a CARD (ASC) or CARD inhibitor of NFκB-activating ligands (Cardinal) and enzymes such as caspase-1 and caspase-5 [6]. It has also been shown that, besides pathogens, antiviral compounds or danger signals of endogenous and xenogenous origins are also able to induce the assembly of these complexes and initiate inflammatory responses [7-9]. NLR proteins as well as their respective molecular complexes are able to modulate intracellular signaling pathways (such as NFκB and MAPK) resulting in altered gene expression signatures [10]. Moreover, caspase-1 is important to cleave pro-IL-1b and pro-IL-18 into their functional forms [11]. These cytokines function as mediators of the immune response by promoting migration and activation of immunocompetent cells. Many of the NLR proteins are involved in inflammatory responses, and some human autoimmune diseases are associated with mutations of defined NLR family members [12].

The expression of several TLR family proteins was studied in human corneal epithelial cells and in related immortalized cell lines [1]. However, no information is available on the presence or activity of NLR family proteins in these cell types. A previous study demonstrated that the upregulation of IL-18 in immortalized human corneal cells by various stimuli (such as lipopolysaccharide [LPS] and phorbol-12-myristate-13-acetate [PMA]) [13] suggests the presence of a functional inflammasome complex in corneal epithelial cells. Here, we report for the first time the cell type-specific steady-state mRNA expression pattern of NLRs and the inflammasome components in human corneal epithelial cells obtained from healthy individuals undergoing photorefractive keratectomy (PRK) treatment and in SV-40 immortalized corneal epithelial cells (HCE-T). We also show the changes of expression of these genes after a low dose UV-B irradiation in HCE-T cells. Furthermore, we have also demonstrated the presence of Nalp-1 and caspase-1 at the protein level in HCE-T cells as well as the functionality of the Nod1/Nod2 system by increasing IκB phosphorylation upon specific agonist stimuli.

## Methods

### Human primary corneal epithelial cells

With proper informed consent and approval of the Human Ethical Committee of the Medical and Health Science Center of the University of Debrecen, human corneal epithelial cells were taken from the eyes of five healthy individuals (two men, three women) who had no history of eye disease. These subjects had myopic or astigmatic symptoms and were undergoing epithelial ablation for photorefractive keratectomy (PRK) with an excimer laser. Just before keratectomy, deepithelialization was performed with a blunt keratome blade after epithelial marking with a 6.0–6.5 mm Hoffer trephine for spherical correction and a 7.5–8.0 mm Hoffer trephine for astigmatic correction centered over the pupil. The epithelium was scraped gently from periphery to center, paying attention not to damage the surface of Bowman’s layer, and the obtained material was put into TRIsol (final volume of 0.5 ml; Invitrogen, San Diego, CA) and then stored at −80 °C until use.

### Human immortalized corneal epithelial cells and THP-1 cells

The SV-40 immortalized human corneal epithelial cell line (HCE-T) was generously provided by Kaoru Araki-Sasaki (Osaka University, School of Medicine, Osaka, Japan) through the Riken Cell Bank. Corneal epithelial cells were cultured in DMEM/F12 cell culture medium containing 200 U/ml penicillin and streptomycin, 5% FBS (Gibco, San Diego, CA), 5% glutamine, 5 μg/ml insulin, 0.5% dimethyl sulfoxide (all from Sigma-Aldrich, St Louis, MO), and 10 ng/ml human epidermal growth factor (Invitrogen). For peptidoglycan treatment, we used 4 μg/ml soluble sonicated peptidoglycan from *Escherichia coli* K12 (PGN-ECndss; Invivogen). THP-1 (Human acute monocytic leukemia cell line) cells were maintained in RPMI 1640 (Invitrogen) supplemented with 5 mM L-glutamine, 100 U/ml penicillin and streptomycin, and 10% FBS. Cells were stimulated with 1 μg/ml lipopolysaccharide (LPS) when indicated (Invivogen).

### Ultraviolet-B treatment of HCE-T cells

When HCE-T cells reached approximately 85%–90% confluency, the growth medium was removed and collected and the cells were irradiated with 30 mJ/cm^2^ dose of UV-B. The UV-B source consisted of a FG15T8 bulb, which produced maximal output in the UV-B range. After irradiation, the cells were cultured further in their conditioned medium for 24 h. Control cells were treated in the same manner except that they were not irradiated. Control and irradiated cells were harvested 6 h and 24 h after UV-B treatment and were stored in 1 ml TRIsol/10^7^ cells at −80 °C. The 3-(4,5-dimethylthiazol-2-yl)-2,5-diphenyltetrazolium bromide assay (MTT) assay to test cell viability after UV-B exposure was performed as described previously [14].

### Isolation of RNA from primary and immortalized human corneal epithelial cells

Total RNA was extracted with TRIsol reagent (Invitrogen, San Diego, CA) and was isolated according to the manufacturer’s instructions. The concentration and homogeneity of the RNA preparations was determined by measuring the absorbance at 260 nm and 280 nm by a spectrophotometer (Nanodrop, ND1000; Bioscience, San Luis Obispo, CA). To avoid contamination by genomic DNA, samples were treated by DNase (Ambion, Austin, TX) and the final RNA preparations were stored at −80 °C.

### TaqMan real time reverse transcription polymerase chain reaction

Reverse transcription of 100–150 ng of total RNA was performed at 42 °C for 1 h and at 72 °C for 5 min by using the Superscript II reverse transcriptase kit (Invitrogen) and random hexamer primers (Invitrogen). Quantitative real-time polymerase chain reaction (PCR) was performed by using ABI PRISM 7900 equipment (Applied Biosystems, Foster City, CA) under the following conditions: 40 cycles at 95 °C for 12 s and 60 °C for 30 s by using TaqMan assays (Applied Biosystems). All PCR reactions were performed in triplicate in 25 μl volumes with one control reaction that contained cDNA but no reverse transcriptase (RT) enzyme. The comparative C_t_ method was used to quantify transcripts, and the expression level was normalized to that of the human housekeeping gene *36B4* or cyclophilin. Normalizing the results to the cyclophilin expression provided results identical to those obtained using *36B4* (data not shown). The oligonucleotide sequences and the specific fluorescence-labeled DNA probes were selected to span exon junctions of the target genes. The following ABI-TaqMan assays were used in the studies: ASC-HS00203118, Caspase-1-Hs00354836, Caspase-5-Hs00362072, Cardinal-Hs00209095, Nalp1-Hs00248187, Nalp3-Hs00366461, Nalp2-Hs215284, Nalp7-Hs00373683, Nalp10-Hs738590, NLRC5-Hs00260008, NLRX1-Hs00226360, Nod1-Hs00196075, and Nod2-Hs00223394.

### Western blot analysis

The treated and untreated cells were washed with ice-cold phosphate-buffered saline and suspended into a lysis buffer with freshly added protease inhibitors. The protein concentration of the samples was determined using the BCA protein assay reagent kit (Pierce, Rockford, IL). Thirty micrograms of total proteins were heated with SDS sample buffer, separated on acrylamide SDS–PAGE gel, and then transferred onto nitrocellulose membranes using wet electroblotting. Membranes were blocked in Tween-Tris-buffered saline (TTBS) containing 5% nonfat milk and were incubated with antibodies that recognized caspase-1 p10 (rabbit polyclonal sc-515; Santa Cruz Biotechnology, Santa Cruz, CA), p-IκB-α (mouse monoclonal; Santa Cruz Biotechnology), Nalp1 and Nalp3 (mouse monoclonal; Alexis Biochemicals, Lausen, Switzerland), and β-actin (monoclonal; Sigma-Aldrich) overnight at 4 °C. Primary antibodies were detected using horseradish peroxidase (HRP)-conjugated secondary antibodies (anti-mouse or anti-rabbit; Amersham, Piscataway, NJ) for 1 h at room temperature. Proteins were visualized by SuperSignal West-Pico peroxide/luminol enhancer solution (Pierce). To detect β-actin expression, we stripped the primary antibody from the nitrocellulose and re-probed the same blots with a second antibody using the same procedure used for the first antibody.

### Determination of secreted cytokine concentrations

The concentration of secreted cytokines in the cell culture supernatant of HCE-T cells was determined by enzyme-linked immunosorbent assay (ELISA). At the time when UV-B treated HCE-T cells were collected for RNA isolation (6 h or 24 h after UV-B irradiation), cell culture supernatants were harvested, centrifuged, and stored at −80 °C until used for cytokine measurements. The concentration of cytokines secreted by HCE-T cells was measured according to the manufacturer’s instruction for IL-1β, IL-6, IL-8, IL-10, IL*-*12 (OPTI-EIA; BD PharMingen, San Diego, CA), and IL-18 (R&D System, Minneapolis, MN). The detection limit of the assays was 10 pg/ml for IL-1β, IL-10, IL-12, and TNF-α, 5 pg/ml for IL-6 and IL-8, and 30 pg/ml for IL-18.

### Statistical analysis

Significant differences between mean values were evaluated using the Student’s *t*-test. Data are presented as mean±SD of the mean.

## Results

### Transcription of *Nalps* and inflammasome components in immortalized and primary corneal epithelial cells

**Figure 1:**
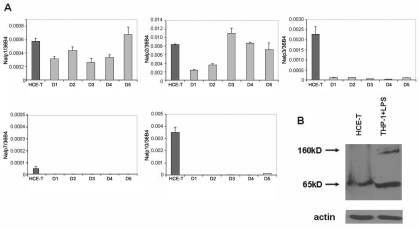
Expression of Nalp family proteins in corneal epithelial cells. The relative expression of Nalp family member mRNA was measured by real time RT–PCR in HCE-T cells and in primary corneal epithelial cells derived from five individuals referred to as donors (D1–D5) as described in Methods. **A**: Relative expression levels of *Nalps* in HCE-T cells (mean values of three independent experiments) and in PRK samples are shown in the charts. Relative gene expression is shown as the ratio of the indicated transcripts relative to *36B4* expression±SD measured in triplicates. **B**: Detection of Nalp1 protein in HCE-T and LPS-treated THP-1 cell lysates by western blotting is illustrated. The uniform loading of the sample amounts was verified by using β-actin antibody.

Using a set of real time reverse transcription polymerase chain reaction (RT–PCR) assays, we measured relative mRNA levels of the members belonging to the Nalp subfamily in immortalized HCE-T cells as well as in primary corneal epithelial cells obtained from five healthy individuals undergoing PRK treatment to correct miophy (Figure 1A.). The expression pattern of *NLR* genes in the different PRK donors (D1-D5) was approximately uniform with the exception of *Nalp2* that showed up to a fivefold difference among the individual primary cell samples. The relative expression of *Nalp1*, the sensory component of Nalp1 inflammasome, was comparable in the immortalized and primary cells. However, the expression of the *Nalp3* sensor was more than 20 fold higher in HCE-T cells than in the PRK samples. *Nalp7* was only detectable at a very low level in HCE-T cells whereas the expression of *Nalp10* was dramatically higher in HCE-T cells than in the PRK samples. Our assays did not detect *Nalp4*, *Nalp6*, or *Nalp12* either in HCE-T or in primary corneal cells, although the corresponding assays were verified by using other cell types that expressed these genes (data not shown).

Using western blotting technique, we aimed to detect the presence of Nalp1 and Nalp3 proteins in the HCE-T cells. As a positive control, we used LPS-treated THP-1 cells that were reported to express these proteins [15]. While we detected a longer (160 kDa) and a shorter (65 kDa) isoform of the Nalp1 protein in the THP-1 cells, we detected only the shorter isoform of Nalp1 in HCE-T cells (Figure 1B). On the other hand, we could not detect the Nalp3 protein in HCE-T cells with the antibody that detected Nalp3 in THP-1 cells (data not shown).

**Figure 2:**
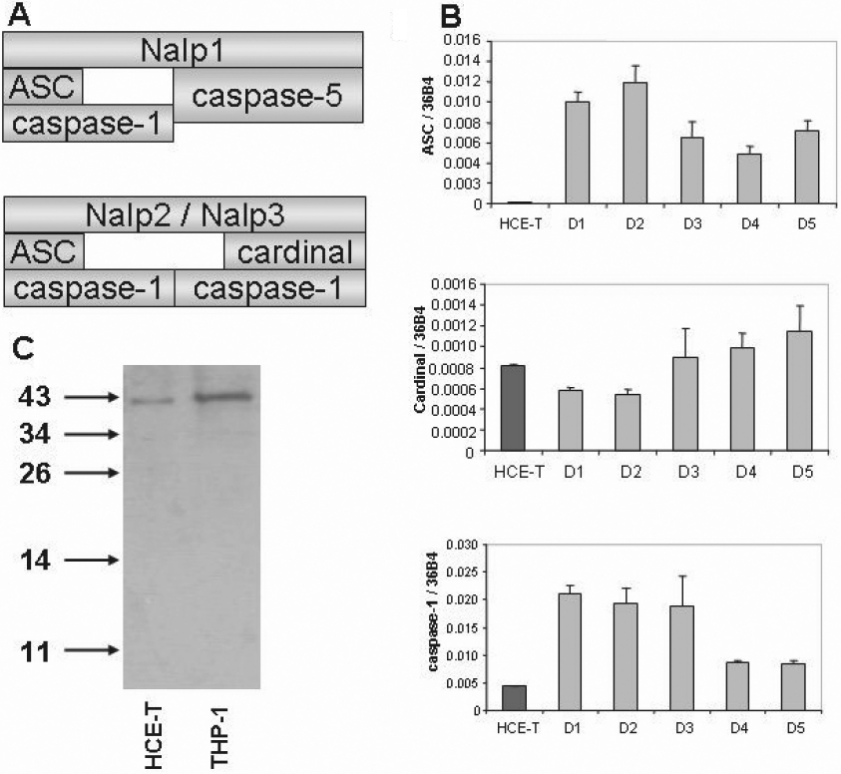
Transcription of inflammasome adaptors and enzymes. The relative expression of adaptors and caspase mRNA was measured by real time RT–PCR in HCE-T cells and in primary corneal epithelial cells derived from five individuals referred to as donors (D1 – D5) as described in Methods. **A**: Composition of the Nalp1 and Nalp2/Nalp3 inflammasomes is diagrammed. **B**: Relative expression levels of Nalp-inflammasome adaptors and enzymes in HCE-T cells (mean values of three independent experiments) and in PRK samples are shown in charts. Relative gene expression is shown as the ratio of the indicated transcripts relative to 3*6B4* expression±SD measured in triplicates. **C**: Detection of caspase-1 protein expression of HCE-T and THP-1 cell lysates by western blotting is illustrated.

Besides the Nalp sensors, inflammasome complexes also contain ASC and Cardinal adaptors as well as caspase-1 and caspase-5 enzymes (Figure 2A). To assess whether the expression levels of these components differ in immortalized and primary corneal cells, we measured their mRNA expression (Figure 2B). The expression level of ASC adaptor was more than two orders of magnitude lower in HCE-T cells as compared to that measured in the PRK samples whereas the expression of Cardinal, a specific adaptor of the Nalp3 inflammasome, was similar in the two cell types. A significantly lower expression of the procaspase-1 enzyme was found in HCE-T cells as compared to that measured in the PRK samples (Figure 2B). However, procaspase-1 was clearly detectable at the protein level in HCE-T cells, while no sign of its activation was observed, as also seen in unstimulated THP-1 cells (Figure 2C). On the other hand, the expression of caspase-5 was below the detection limit in both of these cells even though the assay system was capable to detect this enzyme in other cell types (data not shown).

### Expression of Nod subfamily members in immortalized and primary corneal epithelial cells

**Figure 3:**
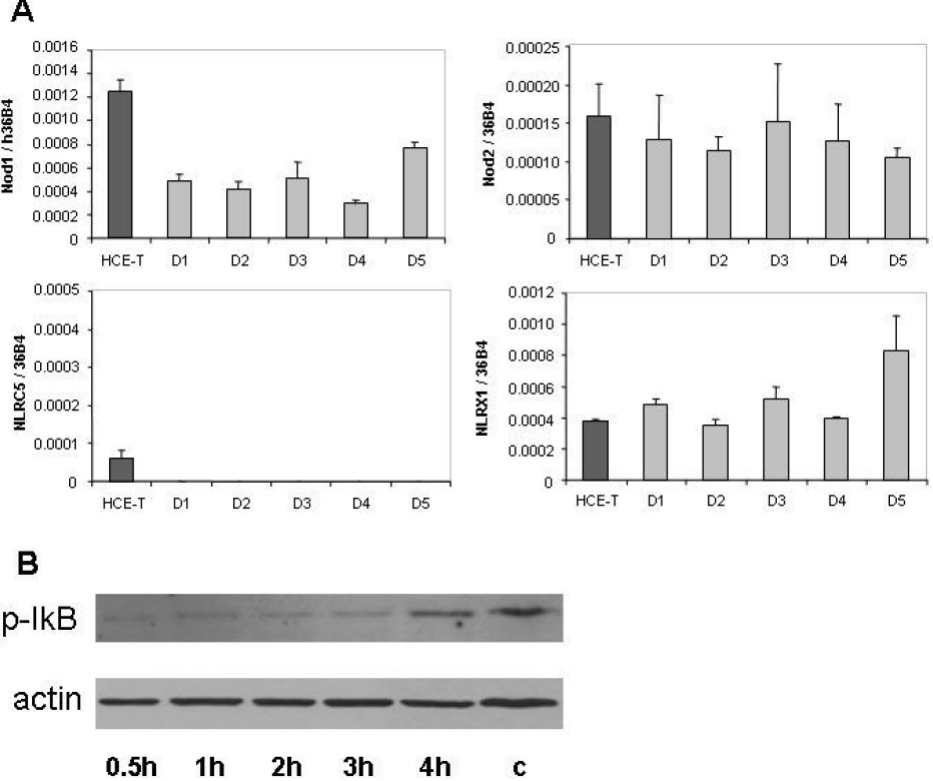
Expression of Nod subtypes in corneal epithelial cells. **A**: The relative expression of *Nod1*, *Nod2*, *NLRC5*, and *NLRX1* mRNA in HCE-T cells and in primary corneal epithelial cells derived from five individuals were measured and documented as described in the legend of Figure 1. **B**: Detection of phosphorylated IκB using western blotting is illustrated. HCE-T cells were treated with 4 μg/ml ultrapure PGN-ECndss for the indicated time periods. The control THP-1 cells (c)  were treated with 1 μg/ml LPS for 4 h. Equal amount of sample loading was verified by detecting β-actin protein expression.

To have an insight into the cell type specific expression of another subfamily of NLRs, we measured the relative mRNA transcription of Nod family members in HCE-T cells and PRK samples (Figure 3A). We found that *Nod1*, *Nod2*, and *NLRX1* were expressed at comparable levels in both cell types with an apparently uniform expression level in the primary corneal cell samples. Similar to that found with the *Nalp1* and *Nalp3* inflammasome components, variation of expression levels of these genes was lower than threefold among the PRK samples. The mRNA of *NLRC5* was undetectable in the primary cells and was expressed at a very low level in HCE-T cells.

Since the expression of *Nod1* and *Nod2* mRNA in HCE-T cells was similar and comparable to that of the primary corneal epithelial cells, we sought to investigate if HCE-T cells are able to recognize and respond a specific agonist. We treated HCE-T cells with ultrapure peptidoglycan (PGN-ECndss, a modified form of peptidoglycan from *E. coli*) that was reported to specifically act via Nod1 or Nod2 without the activation of TLR2. Since the activation of *Nod1* and *Nod2* leads to the activation of NFκB [16] [17] and phosphorylation of IkB-α was demonstrated to be a marker of NFkB activation [18], we studied the appearance of phospho-IkB (pIkB) upon PGN-ECndss treatment of HCE-T cells using western blot technique (Figure 3B). The time-dependent appearance of pIkB after PGN-ECndss treatment implies the presence of functional Nod1 and/or Nod2 proteins in HCE-T cells that are able to recognize the peptidoglycan ligand and trigger the NFκB pathway activation.

### Effect of UV-B irradiation on the transcription of *Nalps* in HCE-T cells

**Figure 4:**
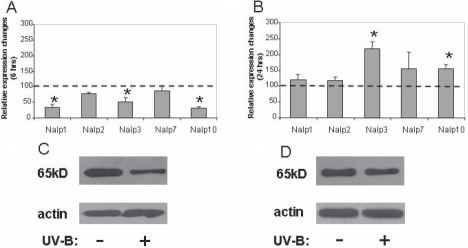
The effect of UV-B treatment on the expression of Nalp subtypes in HCE-T cells. Changes of *Nalp* mRNA expression 6 h (**A**) or 24 h (**B**) after UV-B treatment are shown. The relative expressions of *Nalp1*, *Nalp2*, *Nalp3*, *Nalp7*, and *Nalp10* were measured in 85%–90% confluent HCE-T cells irradiated with 30 mJ/cm^2^ UV-B as described in the legend of Figure 1 and are shown compared to cells incubated in the same way but not irradiated. Mean values and ±SD were calculated from six independent measurements. The asterisk indicates a p<0.005. Changes of Nalp1 protein expression 6 h (**C**) and 24 h (**D**) after UV-B irradiation of HCE-T cells are illustrated. Equal amount of sample loading was verified by detecting β-actin protein expression.

The effect of UV-B irradiation on the transcription of various Nalp family members as well as Nalp1 and Nalp*3* inflammasome components in HCE-T cells was studied at early (6 h) and late (24 h) time points after exposure and was compared to those of non-exposed cells. Relative expression values were normalized to those values obtained at the time of exposure in untreated control samples. Based on the MTT assay performed after 24 h of exposure, the UV-B dose used in these experiments resulted in apoptosis of 10%–13% of total cells. With the exception of Nalp2 and Nalp7, the expression level of the detected Nalps was significantly decreased 6 h after UV-B exposure (Figure 4A). However, as compared to the 6 h samples, their mRNA expression level recovered 24 h after irradiation in such a way that the expression levels of *Nalp3* and *Nalp10* became significantly higher than those measured from non-exposed control cells (Figure 4B). In cases of *Nalp1* and *Nalp3*, we also attempted to study the changes at the protein level. The protein amount of Nap1 showed a marked reduction 6 h after UV-B exposure (Figure 4C) followed by an increase nearly up to the level found in the untreated sample (Figure 4D). However, we could not detect the presence of the Nalp3 protein in any of the samples (data not shown), although the mRNA level of *Nalp3* significantly increased 24 h after exposure.

### Expression of inflammasome adaptors and enzymes in HCE-T cells after UV-B radiation

**Figure 5:**
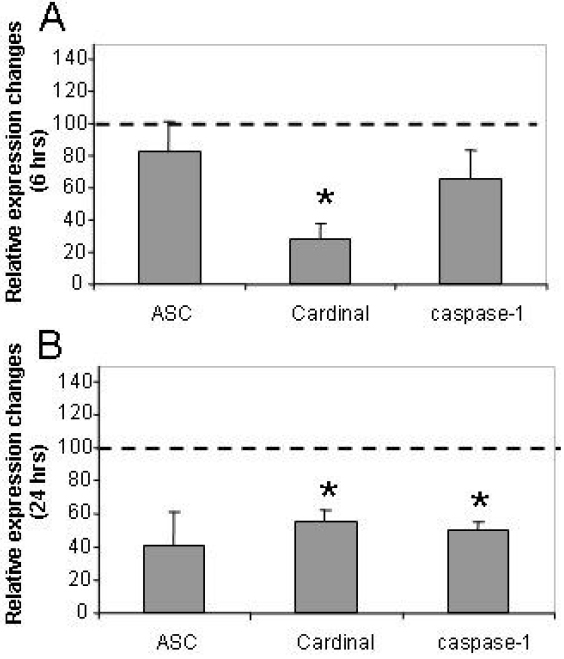
The effect of UV-B treatment on the expression of inflammasome components in HCE-T cells. Changes of inflammasome component mRNA expression 6 h (**A**) or 24 h (**B**) after UV-B treatment are illustrated. The relative expression of *ASC*, *Cardinal*, and *caspase-1* was measured in 85%–90% confluent HCE-T cells irradiated with 30 mJ/cm^2^ UV-B as described in the legend of Figure 1 and is shown as compared to cells incubated in the same way but not irradiated. Mean values and ±SD were calculated from six independent measurements. The asterisk indicates a p<0.005.

We have also determined how the mRNA level of inflammasome components changed upon UV-B irradiation. We found that while all components showed a decreased level of transcription, the changes were significant only for Cardinal as measured 6 h after UV-B exposure (Figure 5A). Twenty-four hours later, the transcription of all components remained lower and the decrease of the caspase-1 level became also significant when compared to the level measured in the non-exposed control cells (Figure 5B).

### Effect of UV-B irradiation on the expression of Nod subtypes in HCE-T cells

**Figure 6:**
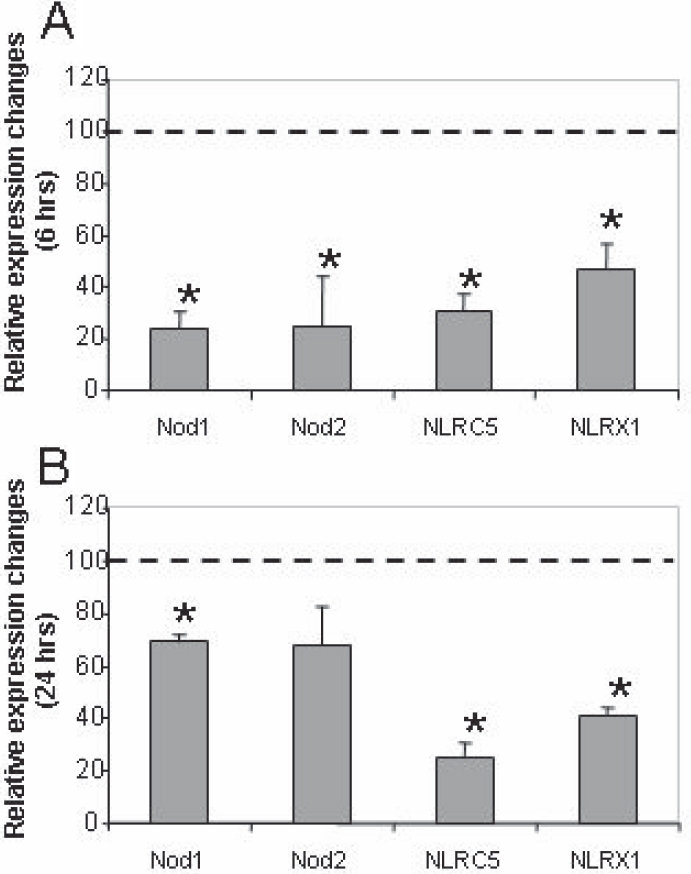
The effect of UV-B treatment on the expression of Nod subtypes in HCE-T cells. Changes of *Nod* mRNA expression 6 h (**A**) or 24 h (**B**) after UV-B treatment are shown. The relative expression of *Nod1*, *Nod2*, *Nod4/Nod27*, and *Nod5/Nod9* was measured in 85%–90% confluent HCE-T cells irradiated with 30 mJ/cm^2^ UV-B as described in the legend of Figure 1 and is shown as compared to cells incubated in the same way but not irradiated. Mean values and ±SD were calculated from six independent measurements. The asterisk indicates a p<0.005.

We also wanted to know how the expression of the Nod subfamily, the other sensor subfamily of the NLR family, would be influenced by the UV-B radiation at early (6 h) and late (24 h) time points. We observed a significant downregulation in the transcription of all the studied Nods 6 h after UV-B radiation (Figure 6A). Surprisingly, after 24 h, although *Nod1* and *Nod2* expression increased compared to the 6 h samples, all the studied *Nod* expression remained low compared to the non-exposed samples (Figure 6B). These results imply that unlike the case of Nalps, there is a general early and long-term downregulation of the Nod sensors after UV-B irradiation.

### Cytokine secretion of HCE-T cells after UV-B irradiation

**Figure 7:**
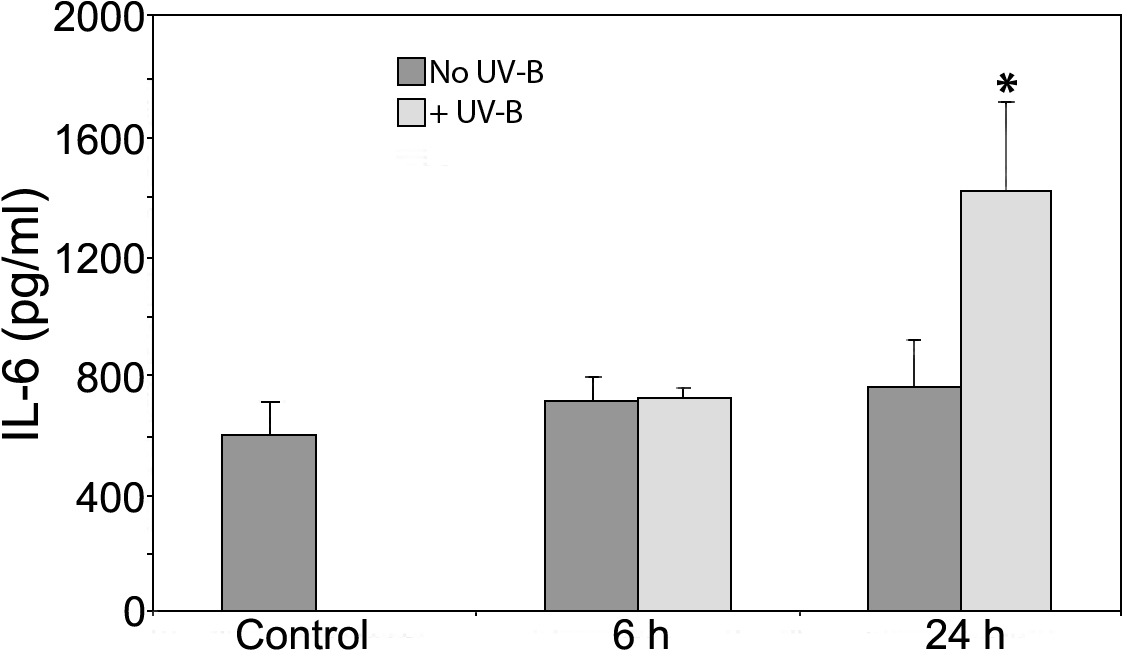
Secretion of IL-6 cytokine induced by UV-B irradiation in HCE-T cells. Cells were irradiated with 30 mJ/cm^2^ UV-B at 85%–90% confluency, and culture supernatants were harvested at the indicated time points. Control cells were treated in the same way but were not irradiated. The concentration of IL-6 cytokine was determined by ELISA.

Based on our results showing a cellular response mediated by NLR sensor molecules, we sought to detect cytokine secretion of HCE-T cells after UV-B exposure. Using ELISA methods, we measured the concentration of IL-1β, IL-6, IL-10, IL-12, IL-18, and TNF-α cytokines that were secreted spontaneously or induced by UV-B in the culture supernatant of HCE-T cells after 6 h and 24 h. The only cytokine we could detect in these samples was IL-6 (Figure 7). It was constitutively secreted by HCE-T cells, and its level increased by only twofold after 24 h of UV-B irradiation. These results show that the cellular response detected by transcriptional changes of NLR expression in HCE-T cells is accompanied by the enhanced secretion of the pro-inflammatory cytokine, IL-6, but not that of the biologically active IL-1β or IL-18.

## Discussion

Although NLR family members are predicted to be an essential part of the innate immunity system, only a few studies have been reported on the expression of NLRs in any kind of epithelial cells [15,19]. Studies on human corneal epithelial cells are usually hampered by the difficulties in isolating and culturing primary cells. Therefore, immortalized corneal epithelial cell lines such as HCE-T have been used instead of in vivo toxicity test performed in rabbit eyes [20-22]. HCE-T cells are widely used as a model for human corneal epithelial research as it retains morphological, biochemical, and functional characteristics of primary corneal epithelia [20]. HCE-T was also successfully used in various studies including gene silencing by RNAi [23] as well as for studying TLR expression [24].

In our studies, we measured the steady-state mRNA expression of *NLR* genes in HCE-T cells and compared it to that measured in primary cells. We found that in contrast to the similar level of constitutive *Nalp1* mRNA expression in primary and HCE-T cells, the expression of *Nalp3* mRNA was much higher in the immortalized cell line than in primary cells. However, we could not detect the expression of Nalp3 protein while we detected the expression of a shorter isoform of Nalp1. The expression of this Nalp1 form that might be a processed form of the 160 kDa protein or a yet to be described isoform that lacks all leucine rich repeats (LRRs) has been found to be present in K562, Jurkat, and THP-1 cells as well as T-lymphocytes and dendritic cells [15]. Although another commercial antibody (Cell signaling polyclonal Nalp1 antibody #4990) has been reported to be able to recognize a protein band with a similar size in different cell types and the p65 level correlated with the mRNA transcription changes upon UV treatment in our study (see below), a nonspecific reactivity of the antibody cannot be ruled out. Verification and characterization of p65 as a short isoform or a proteolytically processed form of Nalp1 requires further studies.

We also found that the transcription of ASC adaptor and caspase-1 enzyme, constituents of the Nalp inflammasomes, are much higher in primary cells than in HCE-T cells. Both ASC and caspase-1 was shown to be involved in apoptosis. The overexpression of caspase-1 may lead to programmed cell death [25]. ASC also has proapoptotic activity [26], and its overexpression inhibits cellular proliferation [27]. Furthermore, it was shown that the promoter region of ASC is often hypermethylated in tumor cells such as breast, lung, and prostate cancer and melanoma [28]. Although HCE-T cells are not considered to be malignant, immortalization may effect the expression and function of proteins that are involved in the death programs. On the other hand, expression pattern of Nod family members appeared to be similar in the two cell types.

Although UV-B irradiation can be potentially harmful, only a few studies have been performed to determine its effect at the molecular level on the immune system of the eye [2]. In our studies, the effect of UV light on the expression of the monitored NLR molecules in the HCE-T cells was different at early (6 h) and late (24 h) time points, and it also resulted in different changes of *Nalps* and *Nods* expression. The UV-B dose used in these experiments resulted in apoptosis of 10%–13% of total cells. Although detached dying cells were washed off the plates and were not used in our measurements, involvement of ongoing apoptotic processes of the attached cells to the observed gene expression can not be excluded. However, these apoptotic processes are not expected to be major contributors to the observed expression changes. Thus, the effect of UV irradiation on the Nalp1 and Nalp3 inflammasome components and on Nod molecules seems to be rather complex. Instead of getting primed for the recognition of altered intracellular structures potentially generated as a consequence of UV irradiation, downregulation of ASC, Cardinal, and caspase-1 might prevent the triggering of the innate immunity system by limiting the available adaptor and enzyme molecules. The uniform downregulation of NLR family members after UV-B treatment is in line with the immunosuppressive effect of the UV light, and it may increase susceptibility toward infection by pathogens. However, as some of the inflammasome components have dramatically altered expression pattern in HCE-T cells compared to the primary corneal cells, studies on primary cells are necessary to define the role of inflammasomes in UV-B mediated corneal processes.

Interestingly, the effect of UV-B on the skin appears to be more extensive and variable than on the cornea by inducing sunburn (inflammation), tanning, and immunosuppression [2,29]. Keratinocytes, constituents of the outer layer of the body and participate in defense mechanisms, were recently reported to produce IL-1β upon UV-B irradiation via the activation of Nalp3-inflammasome [30] as an alternative response to immunosuppression [31]. In our studies, we did not detect IL-1β production by HCE-T cells, but we did observe enhanced IL-6 secretion after 24 h of exposure. IL-6 is a cytokine that is rapidly produced at local tissue sites after disruption of homeostasis due to trauma or infection. It was shown that the NFκB pathway is an important mediator for activation of the *IL-6* gene [32] and that UV-B irradiation induces the NFκB signaling pathway in several cells including the cornea [33,34]. Our results that show IκB phosphorylation upon PGN-ECndss treatment as well as IL-6 inducibility after UV exposure suggest a functional Nod1/Nod2-initiated NFκB pathway in HCE-T cells. The exact role of IL-6 in the eye is controversial as it is able to initiate inflammation but also may have anti-inflammatory effects. It has also been described that IL-6 reduces the level of pro-inflammatory mediators and induces glucocorticoid synthesis and the production of natural antagonists of IL-1α [35]. IL-6 was shown to be produced by corneal epithelial cells upon viral infection and acts as a chemoattractant since it recruits neutrophil granulocytes from the stroma of the cornea [36]. Thus, UV-B induced IL-6 production may modify the distribution of inflammatory cells in the cornea as it has been observed in the skin [37].

The low constitutive secretion and the relatively weak induction of IL-6 production induced by UV-B together with the lack of other inflammatory cytokine production (IL-1β, TNF-α, and IL-12) suggest a general immunosilenced state of HCE-T cells. The unresponsive nature of HCE-T cells has also been demonstrated by the lack of IL-6, IL-8, and human β-defensin-2 secretion to activation by LPS also indicated the unresponsive nature of HCE-T cells. [24]. The intracellular location of TLR2 and TLR4 in human corneal epithelial cells was reported to prevent innate immune responses and thus may contribute to maintain an immunosilent environment at the ocular mucosal epithelium. Nevertheless, it is also possible that the low level of IL-6 may act as a trigger to exert the inflammatory response of the stromal cells, the primary cause of photokeratitis [38].

While the different expression levels of inflammasome components in primary corneal cells and HCE-T cells suggest that the immortalized cells may behave differently to Nalp ligands, the similar expression pattern of Nod sensors in the two cell types and the functional Nod1/Nod2-dependent NFκB signaling pathway may render the HCE-T cells better suitable for model studies of Nod-signalosome. It was reported that the mutation of human *Nod2* causes an autosomal dominant form of uveitis that is characteristic of Blau syndrome [39]. Moreover, muramyl dipeptide (MDP) was recently reported to generate IL-1β within the eye of mice, and this process is dependent on Nod2 and caspase-1 [40]. However, these studies did not focus on the involvement of epithelial cells in these processes. For this reason, finding a good model system of corneal epithelial cells for Nod1/Nod2 studies in the eye is becoming particularly important.
